# Prognostic value of metabolic tumor volume on [^18^F]FDG PET/CT in addition to the TNM classification system of locally advanced non-small cell lung cancer

**DOI:** 10.1186/s40644-024-00811-7

**Published:** 2024-12-21

**Authors:** Alexander Brose, Isabelle Miederer, Jochem König, Eleni Gkika, Jörg Sahlmann, Tanja Schimek-Jasch, Mathias Schreckenberger, Ursula Nestle, Jutta Kappes, Matthias Miederer

**Affiliations:** 1 Department of Translational Imaging in Oncology, National Center for Tumor Diseases (NCT/UCC) Dresden, Medical Faculty and University Hospital Carl Gustav Carus, TUD Dresden University of Technology, Fetscherstraße 74, Dresden, 01307 Germany; 2 German Cancer Research Center (DKFZ), Heidelberg, Germany; 3 Helmholtz-Zentrum Dresden-Rossendorf (HZDR), Dresden, Germany; 4 Department of Diagnostic and Interventional Radiology, University Hospital Giessen, Justus Liebig University, Klinikstrasse 33, Giessen, 35392 Germany; 5 Member of the German Center for Lung Research (DZL), Giessen, Germany; 6 Department of Nuclear Medicine, University Medical Center Mainz, Johannes Gutenberg-University Mainz, Mainz, Germany; 7 Institute of Medical Biostatistics, Epidemiology and Informatics (IMBEI), University Medical Center Mainz, Johannes Gutenberg-University Mainz, Mainz, Germany; 8 Department of Radiation Oncology, University Hospital Bonn, Bonn, Germany; 9 Department of Radiation Oncology, University Hospital Freiburg, Freiburg, Germany; 10 Institute of Medical Biometry and Statistics (IMBI), Faculty of Medicine, University Medical Center Freiburg, Freiburg, Germany; 11 Department of Radiation Oncology, Kliniken Maria Hilf, Mönchengladbach, Germany; 12 Department of Pulmonary Medicine, Theresienkrankenhaus, Mannheim, Germany; 13 Department of Internal Medicine/ Pulmonary Medicine, Catholic Hospital Koblenz-Montabaur, Koblenz, Germany

**Keywords:** PET/CT, Lung cancer, NSCLC, Metabolic tumor volume, TNM, Prognosis

## Abstract

**Purpose:**

Staging of non-small cell lung cancer (NSCLC) is commonly based on [^18^F]FDG PET/CT, in particular to exclude distant metastases and guide local therapy approaches like resection and radiotherapy. Although it is hoped that PET/CT will increase the value of primary staging compared to conventional imaging, it is generally limited to the characterization of TNM. The first aim of this study was to evaluate the PET parameter metabolic tumor volume (MTV) above liver background uptake as a prognostic marker in lung cancer. The second aim was to investigate the possibility of incorporating MTV into the TNM classification system for disease prognosis in locally advanced NSCLC treated with chemoradiotherapy.

**Methods:**

Retrospective evaluation of 235 patients with histologically proven, locally advanced NSCLC from the multi-centre randomized clinical PETPLAN trial and a clinical cohort from a hospital registry. The PET parameters SUVmax, SULpeak, MTV and TLG above liver background uptake were determined. Kaplan-Meier curves and stratified Cox proportional hazard regression models were used to investigate the prognostic value of PET parameters and TNM along with clinical variables. Subgroup analyses were performed to compare hazard ratios according to TNM, MTV, and the two variables combined.

**Results:**

In the multivariable Cox regression analysis, MTV was associated with significantly worse overall survival independent of stage and other prognostic variables. In locally advanced disease stages treated with chemoradiotherapy, higher MTV was significantly associated with worse survival (median 17 vs. 32 months). Using simple cut-off values (45 ml for stage IIIa, 48 ml for stage IIIb, and 105 ml for stage IIIc), MTV was able to further predict differences in survival for stages IIIa-c. The combination of TNM and MTV staging system showed better discrimination for overall survival in locally advanced disease stages, compared to TNM alone.

**Conclusion:**

Higher metabolic tumor volume is significantly associated with worse overall survival and combined with TNM staging, it provides more precise information about the disease prognosis in locally advanced NSCLC treated with chemoradiotherapy compared to TNM alone. As a PET parameter with volumetric information, MTV represents a useful addition to TNM.

**Supplementary Information:**

The online version contains supplementary material available at 10.1186/s40644-024-00811-7.

## Background

Current guidelines for non-small cell lung cancer (NSCLC) recommend fluorine-18-fluorodeoxyglucose positron emission tomography/computed tomography ([^18^F]FDG PET/CT) as the most accurate imaging modality for staging [1]. Its visual evaluation and correct interpretation are crucial for guiding therapy, especially in locally advanced disease [2]. Current therapy approaches in unresectable stages typically involve a combination of platinum-based concurrent chemoradiotherapy (CRT), targeted therapy based on mutational status and immunotherapy with antibodies for the programmed cell death 1 (PD-1) or -ligand (PD-L1), e.g. durvalumab [1, 3–5]. PET/CT has proven to be of utmost importance in target volume definition of modern radiotherapy [6] and could possibly guide administration regimen of immunotherapeutic agents [7]. However, within UICC substages, there are substantial differences in individual disease prognosis. Among other factors like demographics and molecular tumor patterns, this also depends decisively on the tumor burden [7–9]. These considerations on the three-dimensional volume of the tumor spread have not yet been implemented in the TNM system, which rather describes a hypothetical sequential spread of the tumor from localized to systemic disease and only accounts for tumor size of the primary tumor lesion [1]. However, in PET/CT imaging, it is possible to derive volumetric metabolic parameters, i.e. the Metabolic Tumor Volume (MTV) and Total Lesion Glycolysis (TLG), which are used to classify disease extent and guide therapeutic management [8–14]. These metabolic parameters lead to promising results in estimating disease prognosis in a lot of tumor entities along with NSCLC [15–24]. Although most studies indicate the superiority for MTV over traditional PET parameters, e.g. maximum standardized uptake value (SUV_max_) [7, 21, 25–27], two major problems regarding its clinical implementation remain:

1. There is no consistent definition of the method used to determine MTV. In estimating prognosis, absolute or relative thresholds are commonly used [12, 15–18, 28], as well as gradient-based strategies - often as a by-product of radiation therapy planning [19–21, 29, 30]. In response evaluation and relapse assessment, adaptive background normalization techniques, which adjust the threshold on a case-by-case basis, e.g. physiological uptake of the liver parenchyma, are more commonly used [31–34].
2. Establishing cut-off values for assessing prognosis is challenging, since they might depend on varying factors like injected activity, time interval between [^18^F]FDG injection and scan, blood glucose level, and image reconstruction among others. A broad range of suggestions in literature exists, such as dichotomizing by median or percentiles [15, 20, 21, 24, 26, 27, 29], sequential log rank testing [19] or ROC curves [16, 25, 30, 35]. Few studies investigated the potential of MTV for supplementing TNM [17, 19, 24, 36], but none regarding individual tumor uptake above the physiological background threshold as suggested in the “Positron Emission Tomography Response Criteria In Solid Tumors” (PERCIST) [32].

The primary objective of this study was to investigate the prognostic value of metabolic tumor volume above an individual threshold of liver background uptake in patients with locally advanced NSCLC. As second objective, we tested its potential to expand traditional TNM staging for estimation of disease prognosis in patients receiving chemoradiotherapy.

## Methods

### Data collection and study design

**Fig. 1:**
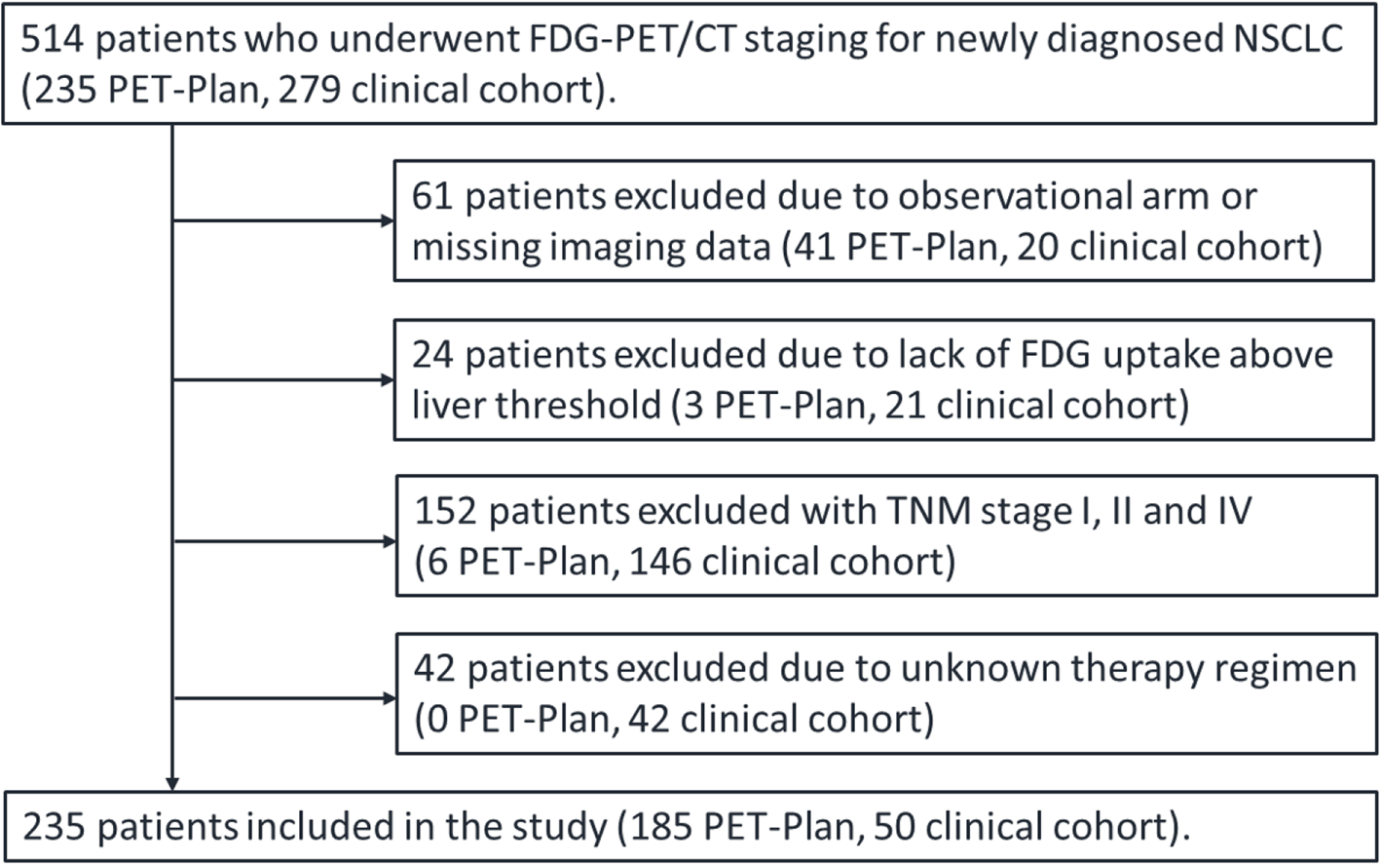
Flow diagram of patient selection

This retrospective analysis included 235 patients with histologically proven NSCLC and UICC stages IIIa-c. All patients underwent [^18^F]FDG-PET/CT imaging for staging, either at a tertiary referral hospital^c^ between January 2017 and December 2019 or as part of the multicenter randomized controlled trial PETPLAN (NCT00697333) between 2010 and 2016. Baseline scans and the variables age, gender, histology, and treatment of the clinical cohort were obtained from local cancer registry databases (UCT Mainz, Lung Cancer Center Koblenz) and follow-up information was supplemented by the documentation of the Federal State Tumor Registry (Krebsregister Rheinland-Pfalz). UICC stages (8th edition) for the clinical cohort were obtained from the interdisciplinary tumor board documentation and manually reclassified for the trial cohort based on the changes for the T-stage from 7th to 8th edition. Data collection and exclusion criteria of the trial cohort have been described previously [6]. In the clinical cohort, patients received treatment according to the decision of the tumor board consensus and either underwent radical surgery or radiotherapy with concurrent chemotherapy consisting of a platinum-based doublet, according to clinical guidelines. In the trial group, patients received dose-escalated radiotherapy (60–74 Gy, 2 Gy per fraction), planned to the respective target volumes and applied with concurrent platinum-based chemotherapy [6]. Treatment duration was 6 to 8 weeks, depending on the total dose prescribed. A total of 279 from the 514 patients originally identified were excluded for lack of baseline imaging, non-FDG-avid tumor, incomplete information on demographics, therapy or follow-up, concurrent cancer diagnosis, or no locally advanced disease stage (Fig. 1). The primary endpoint was overall survival (OS), defined as the time between baseline PET/CT and the date of death by any cause or date of last contact. Explorative analyses included the identification of the most prognostic PET parameter and transformations of the parameter MTV in nested Cox models. This study was approved by the Institutional Ethics Committee in addition to the main trial for the PET Plan cohort (ARO-2009-09) and approval was waived by the competent Ethic committee for the clinical cohort.

### Image acquisition

Diagnostic whole-body PET/CT images were acquired ≥ 60 min post injection of the diagnostic reference activity of [^18^F]FDG in the fasting patient with either full-dose contrast enhanced or low dose CT (Philips Gemini TF 16, GE Discovery 600; Philips Allegro Body, Dual GS, Gemini TF 16, TF 64, TF Big Bore, GXL 6, Guardian Body; Siemens Biograph HiRes, mCT 40, mCT 64, mCT 128, Biograph Truepoint TrueV, Emotion Duo) [6]. All PET scanners and local protocols from more than 20 different institutions have been subject to central quality assurance and calibration by phantom measurements to harmonize imaging data [37]. State-of-the-art iterative reconstruction algorithms were used after correction for attenuation and scatter, decay and randoms.

### Delineation of PET parameters

Computer-aided quantification of PET parameters was performed using the Software Hybrid 3D TumorFinder v2.2 (Hermes Medical Solutions, Stockholm, Sweden) as previously published [33]. PET parameters (MTV, TLG, SUV_max_, SUL_peak_) above liver background uptake were generated semi-automatically, following the threshold definition in PERCIST: a reference volume of interest (VOI) with a diameter of approximately 3 cm was set in the right liver lobe [32]. The software marked every lesion in the patient consisting of more than 3 adjacent voxels showing FDG uptake higher than 1.5*SUL_mean_ + 2*SD of the reference VOI. Only lesions with a morphological correlate were rated as positive, whereas physiological uptake and benign lesions were manually excluded. Number of thoracic lesions was counted and MTV was defined as the sum of the volumes for every single lesion. Total Lesion Glycolysis (TLG) was defined as MTV*SUL_mean_.

### Statistical analyses

Descriptive statistics are presented as mean and standard deviation, median and interquartile range or counts and percentages, and survival times with estimated median and 95% confidence interval. Differences between subgroups were statistically tested using Pearson’s Chi-squared test for categorical variables and Mann-Whitney U test for interval scaled variables. Kaplan-Meier survival curves with log rank testing were used to assess overall survival. All variables were tested in univariable Cox proportional hazards survival models and significant prognostic variables were included in the multivariable model. Since the data did not meet the assumption of a normal distribution, several transformations for MTV were tested for best fit using log likelihood ratio chi squared statistics comparing in nested models. Eventually, a cubic root transformation MTV(r), resulting in the radius of MTV (= r = $\:\sqrt[3]{\frac{3MTVwb}{4\pi\:}}$) was used for the multivariable cox regression. Receiver operating characteristics (ROC) curve analysis with Youden-Index was used to determine optimal cut-off values for MTV [16–19]. The significance level was defined as α = 0.05. All analyses were performed using SPSS v29 (IBM, Armonk, NY, USA) and R software (R Foundation, Vienna, Austria). Study concept and manuscript preparation adhered to the TRIPOD statement [38].

## Results

### Patient characteristics

Two hundred and thirty-five patients were included in the final analysis (Fig. 1). Mean age was 66 years (± 9 years) and 26% were women. The variables histology, treatment, stage and the PET parameters differed significantly between the clinical and the trial cohort (*p* < 0.05); age, gender and number of lesions did not (*p* > 0.05) (Table 1). Median OS was 28 months (95% CI 20–35) with 1-, 3-, and 5-year survival rates of 70%, 43%, and 28%. Median OS of stage IIIa, IIIb and IIIc was 40, 29, and 13 months, respectively. A total of 140 patients died during the follow-up period. Median follow up of the 95 surviving patients was 34 months. There was no statistically significant difference in OS between both cohorts when adjusted to TNM stage. We observed a higher survival rate in patients with adenocarcinoma (AC) compared to patients with squamous cell carcinoma (SCC) with median OS of 42 vs. 24 months (logrank test *p* = 0.004). Patients who underwent surgical treatment had better median OS, compared to chemoradiotherapy with 51 vs. 26 months (logrank test *p* = 0.027, data not shown).

**Table 1:** Characteristics of study patients

Variable	All patients	Clinical cohort	Trial cohort	*p *value
Patients, n (%)	235 (100)	50 (21.3)	185 (78.7)	
Age, mean (±SD)	66.1 (±8.5)	67.6 (±8.8)	65.7 (±8.4)	.266^a^
Gender, n (%)				.356^b^
Female	62 (26.4)	14 (28.0)	48 (25.9)	
Male	173 (73.6)	36 (72.0)	137 (74.1)	
Histology, n (%)				< .001^b^
SCC	124 (52.8)	17 (34.0)	107 (57.8)	
AC	76 (32.3)	21 (42.0)	55 (29.8)	
NOS/ others	35 (14.9)	12 (24.0)	23 (12.4)	
Treatment, n (%)				< .001^b^
Chemoradiotherapy	211 (89.8)	26 (52.0)	185 (100)	
Surgery	24 (10.2)	24 (48.0)		
UICC stage (8^th^edition), n (%)				< .001^b^
IIIa	67 (28.5)	23 (46.0)	44 (23.8)	
IIIb	113 (48.1)	22 (44.0)	91 (49.2)	
IIIc	55 (23.4)	5 (10.0)	50 (27.0)	
PET parameter, median (IQR)				
MTV	73.3 (29 - 129)	64.9 (21 - 96)	76.4 (33 - 135)	< .001^c^
TLG	385.1 (136 - 716)	276.3 (107 - 601)	414.9 (162 - 832)	< .001^c^
SULpeak	10.0 (7 - 13)	9.0 (7 - 11)	10.2 (8 - 13)	< .001^c^
SUVmax	15.8 (12 - 20)	14.6 (12 - 17)	16.8 (13 - 20)	.004^c^
n° of lesions, median (IQR)	2 (1 - 4)	3 (1 - 5)	2 (1 - 4)	0.204^a^

*n* counts, *SD* Standard deviation, *SCC* Squamous cell carcinoma, *AC* Adenocarcinoma, *NOS* Not otherwise specified, *IQR* Interquartile range, *MTV* Metabolic tumor volume, *TLG* Total lesion glycolysis, *SUL* Standardized uptake value corrected for lean body mass, *SUV* Standardized uptake value, *n°* number

^a^Mann-Whitney U-test

^b^Chi-squared-test

^c^t-test

### Cox proportional hazards regression analyses

In the univariable analyses, the variables histology, treatment, stage, and the PET parameters MTV and TLG were significant prognostic factors for OS (Table 2). The PET parameters SUVmax, SULpeak, SULmean, and the variables gender and number of PET-positive lesions were not prognostic of OS. The cubic root transformation MTV(r) resulted in better model fit compared to linear, log and squares transform in Cox models. Multivariate analysis was then performed with all variables that were predictive for OS in univariate analyses and MTV(r) (Table 3). The variables age, stage, and MTV(r) were significant predictors for OS when adjusted to each other and for cohort, histology, and treatment. The cubic root transformation of MTV showed a unit hazard ratio of 1.27. In other words, if the radius of the tumor volume increases by 1 cm in size, the hazard rate for an unfavorable outcome increases by 27%. Additionally, multivariable Cox Proportional Hazards Regression Analyses including clinical variables were performed for raw MTV and TLG (Supplementary Tables 1 and 2). Raw MTV and TLG both were significant predictors for OS when adjusted to the aforementioned variables.

**Table 2:** Univariable Cox Proportional Hazards Regression Analyses for Overall Survival

Variables	All patients				Clinical cohort				Trial cohort			
	HR	95%	CI	*p *value	HR	95%	CI	*p *value	HR	95%	CI	*p *value
Cohort^a^	1.526	2.341	0.994	0.053								
Gender												
Female	reference category				reference category				reference category			
Male	1.233	0.837	1.816	0.289	2.065	0.773	5.517	0.148	1.089	0.714	1.662	0.692
Age	1.015	0.996	1.035	0.125	0.987	0.941	1.035	0.582	**1.027**	1.005	1.049	0.017
Histology				0.010				0.003				0.436
AC	reference category				reference category				reference category			
SCC	**1.805**	1.209	2.696	0.004	**5.214**	1.777	15.302	0.003	1.325	0.859	2.044	0.203
NOS/ others	**1.819**	1.092	3.028	0.022	**6.228**	2.037	19.048	0.001	1.268	0.696	2.31	0.437
Treatment												
Surgery	reference category				reference category							
Chemoradiotherapy	**2.037**	1.068	3.884	0.031	1.786	0.806	3.955	0.153				
UICC stage				< 0.001				0.031				0.005
IIIa	reference category				reference category				reference category			
IIIb	1.519	0.999	2.310	0.050	0.949	0.410	2.201	0.904	**1.686**	1.015	2.800	0.044
IIIc	**2.612**	1.625	4.200	< 0.001	**4.218**	1.331	13.363	0.014	**2.499**	1.432	4.361	0.001
PET parameters												
MTV(r)	**1.419**	1.168	1.724	< 0.001	**1.676**	1.116	2.516	0.013	**1.316**	1.049	1.651	0.018
MTV	**1.003**	1.001	1.004	< 0.001	**1.004**	1.001	1.007	0.011	**1.002**	1.000	1.004	0.014
TLG	**1.000**	1.000	1.000	0.005	**1.000**	1.000	1.001	0.024	1.000	1.000	1.001	0.074
SULpeak	1.011	0.972	1.051	0.592	1.073	0.99	1.163	0.084	0.989	0.944	1.035	0.632
SUVmax	1.005	0.980	1.029	0.714	1.042	0.991	1.096	0.108	0.992	0.963	1.021	0.565
n° of lesions	1.001	0.951	1.055	0.957	1.014	0.948	1.084	0.682	1.017	0.928	1.116	0.716

a, trial cohort compared to clinical cohort. Bold values denote statistical significance

*Abbreviations:* *HR* Hazard Ratio, *CI* Confidence Interval, *NOS* Not otherwise specified, *PET* Positron emission tomography, *MTV(r)* Radius of metabolic tumor volume, *TLG* Total lesion glycolysis, *SULpeak* Peak standardized uptake value corrected for lean body mass, *SUVmax* Maximum standardized uptake value, *n°* number

**Table 3:** Multivariable Cox proportional Hazards Regression Analysis for Overall Survival

Variables	All patients				Clinical cohort				Trial cohort			
	HR	95%	CI	*p* value	HR	95%	CI	*p* value	HR	95%	CI	*p* value
Cohort^a^	1.058	0.612	1.828	0.840								
Age	**1.022**	1.002	1.043	0.031	0.956	0.906	1.008	0.098	**1.034**	1.011	1.058	0.004
Histology				0.056				0.003				0.441
AC	reference category				reference category				reference category			
SCC	**1.618**	1.073	2.439	0.022	**8.116**	2.320	28.391	0.001	1.328	0.859	2.052	0.201
NOS/ others	1.628	0.959	2.764	0.071	**9.009**	2.246	36.141	0.002	1.232	0.67	2.267	0.502
Treatment												
Surgery	reference category				reference category							
Chemoradiotherapy	1.393	0.620	3.131	0.422	1.433	0.618	3.322	0.402				
UICC stage				0.003				< 0.001				0.007
IIIa	reference category				reference category				reference category			
IIIb	1.456	0.948	2.237	0.086	0.909	0.368	2.245	0.836	**1.855**	1.104	3.116	0.020
IIIc	**2.372**	1.432	3.930	< 0.001	**13.588**	3.349	55.124	< 0.001	**2.613**	1.457	4.686	0.001
MTV(r)	**1.266**	1.025	1.562	0.028	1.132	0.618	2.074	0.688	1.211	0.956	1.534	0.112

a, trial cohort compared to clinical cohort. Bold values denote statistical significance

*Abbreviations:* *HR* Hazard Ratio, *CI* Confidence Interval, *NOS* Not otherwise specified, *MTV(r)* Radius of metabolic tumor volume

### PET parameters

**Fig. 2:**
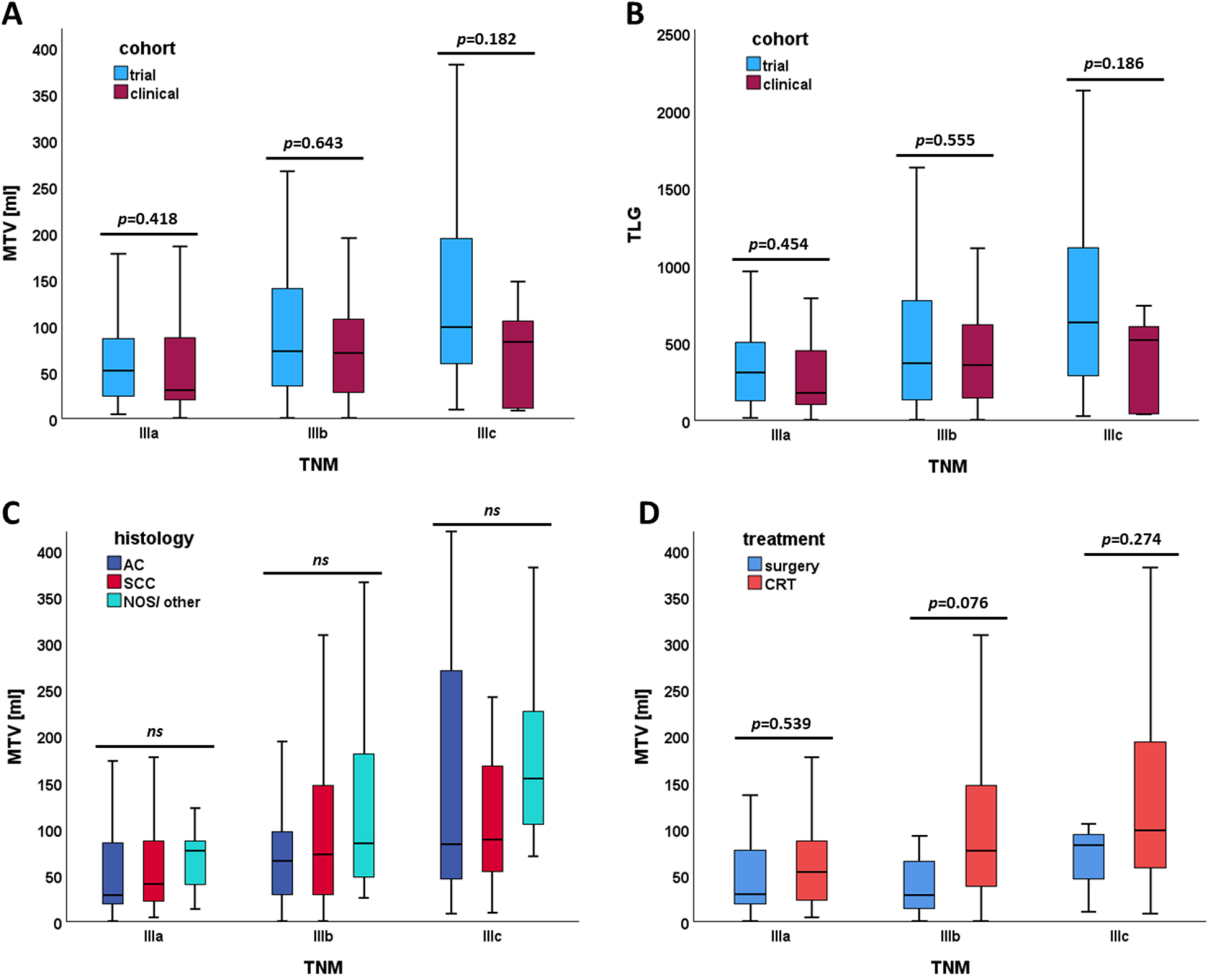
Boxplots of metabolic tumor volume and total lesion glycolysis based on UICC substages according to (**A**, **B**) cohort, (**C**) histology, and (**D**) treatment. Abbreviations: MTV, metabolic tumor volume; TLG, total lesion glycolysis; AC, adenocarcinoma; SCC, squamous cell carcinoma; NOS, not otherwise specified; CRT, chemoradiotherapy; *ns*, not significant (including differences between means of AC vs. SCC, AC vs. NOS/ other, and SCC vs. NOS/ other)

The volumetric parameters MTV and TLG showed significantly higher values in more advanced stages (Fig. 2A, B). In contrast, means of SULpeak, SUVmax, and SULmean did not differ between stages (*p* > 0.05). Patients who underwent chemoradiotherapy had significantly higher mean MTV, compared to surgical candidates (103.8ml vs. 51.2ml, *p* < 0.001) (Fig. 2D). Mean MTV between histological subtypes did not differ significantly between SCC and AC (93.6ml vs. 94.3ml, *p* = 0.235) (Fig. 2C).

### MTV in locally advanced NSCLC treated with chemoradiotherapy

**Fig. 3:**
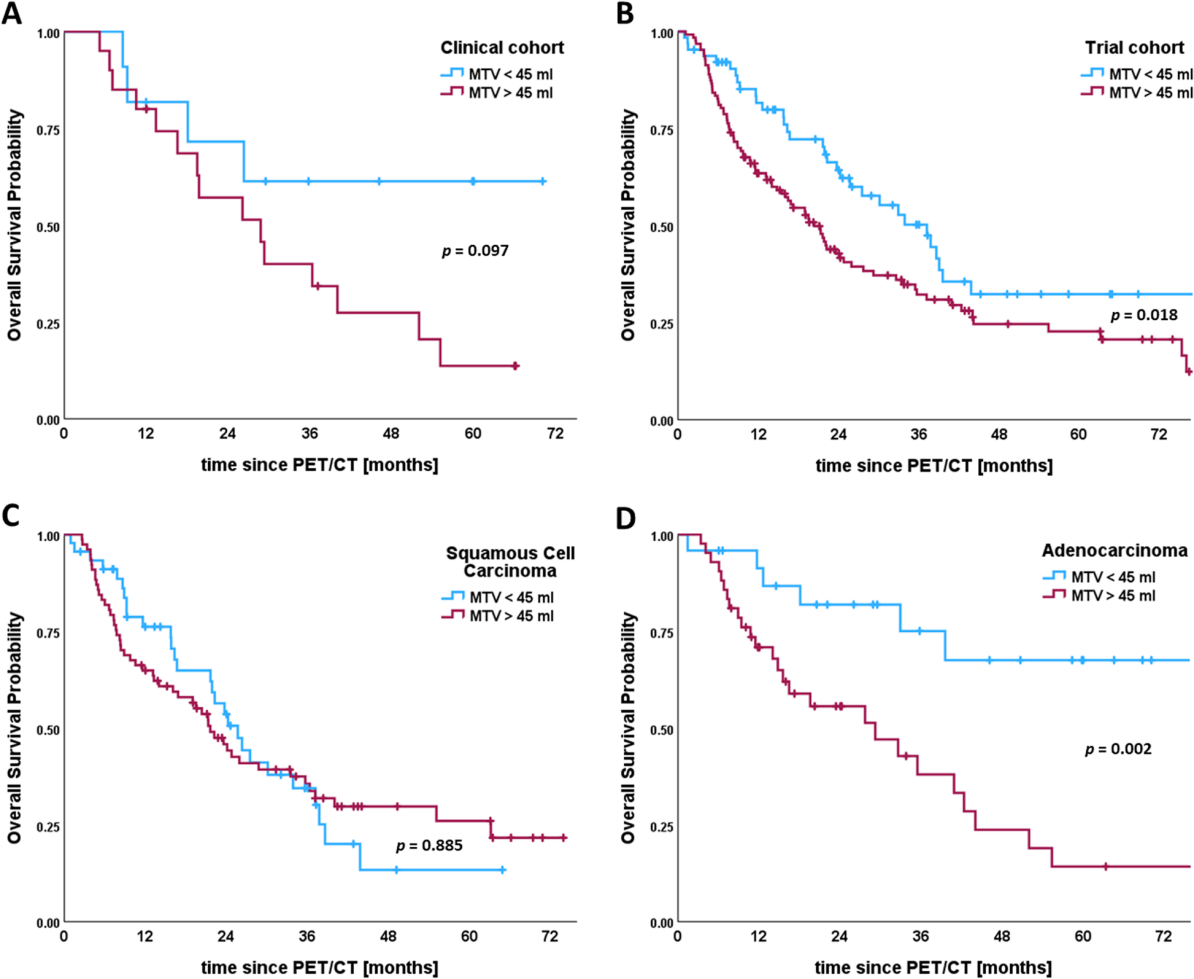
Kaplan-Meier plots stratified by metabolic tumor volume for all patients with locally advanced non-small cell lung cancer receiving chemoradiotherapy (*n* = 211). Plots are divided by cohorts into (**A**) clinical cohort and (**B**) trial cohort, and by histology for patients with (**C**) squamous cell carcinoma and (**D**) adenocarcinoma. **A** Median survival in the clinical cohort was not reached for patients with metabolic tumor volumes below 45 ml, and 29 months for tumor volumes above 45 ml (logrank *p* = 0.097). **B** Median survival in the trial cohort was 37 months (95% CI 27–47) vs. 20 months (95% CI 16–25) (logrank test *p* = 0.018). **C** Median survival for patients with squamous cell carcinoma was 26 months (95% CI 20–31) vs. 22 months (95% CI 17–26) for tumor volumes below and above 45 ml, respectively (logrank *p* = 0.885). **D** Median survival for patients with adenocarcinoma was not reached for the lower tumor volume group, and 29 months (95% CI 11–47) for the higher tumor volume group (logrank test *p* = 0.002). Vertical lines indicate censoring

**Fig. 4:**
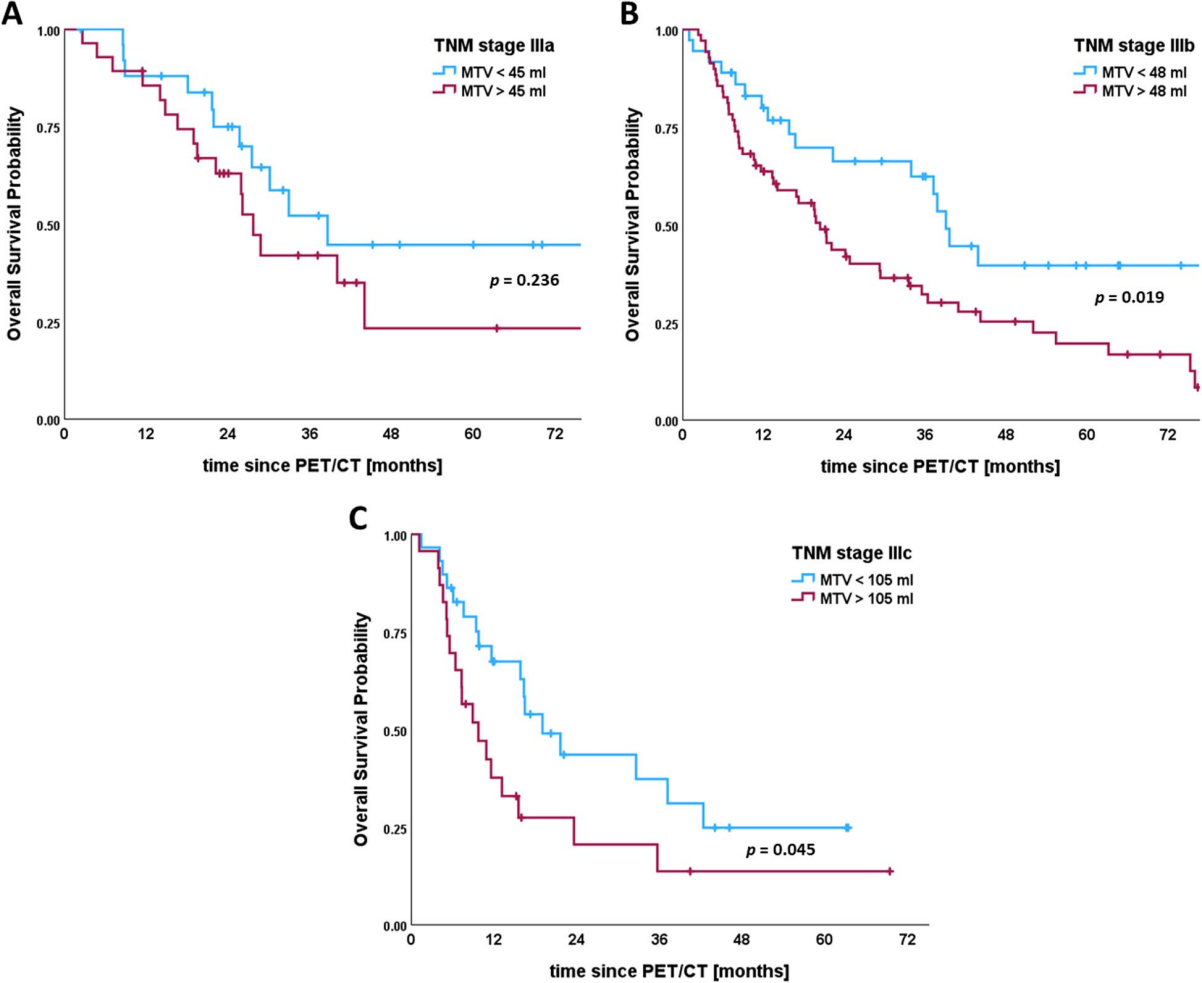
Kaplan-Meier plots for optimal cut-off values when combining metabolic tumor volume and UICC stages IIIa-c in patients with locally advanced non-small cell lung cancer receiving chemoradiotherapy. **A** Median overall survival for stage IIIa was 39 months (95% CI 24–53) for tumor volumes below 45 ml vs. 28 months (95% CI 24–31) for tumor volumes above 45 ml (logrank test *p* = 0.236). **B** Median overall survival for stage IIIb was 39 months (95% CI 36–42) for tumor volumes below 48 ml vs. 20 months (95% CI 15–25) for tumor volumes above 48 ml (logrank test *p* = 0.019). **C** Median overall survival for stage IIIc was 19 months (95% CI 12–26) for tumor volumes below 105 ml vs. 10 months (95% CI 5–15) for tumor volumes above 105 ml (logrank *p* = 0.045). Vertical lines indicate censoring

In further analyses we focused on the subset of 211 patients who underwent platinum-based, concurrent chemoradiotherapy. When stratified by a cut-off value of 45 ml, higher MTV shows significantly worse OS in both, the trial and clinical cohort (Fig. 3A, B). Comparing histological subtypes, a significant difference in OS for the same cut-off value can be found in patients with adenocarcinoma (Fig. 3D), but not with squamous cell carcinoma (Fig. 3C). Using ROC-curve analyses, we identified individual MTV cut-off values for TNM stages IIIa-c. When applied to each specific stage, they allowed for further estimating survival curves of the UICC stages IIIa (cut-off 45 ml), IIIb (cut-off 48 ml), and IIIc (cut-off 105 ml). We found significantly different median OS in stages IIIb and IIIc, and a clear trend of higher MTV being associated with worse OS for all three substages (Fig. 4).

### Combined risk stratification (TNM + MTV) of patients treated with RCT

**Fig. 5:**
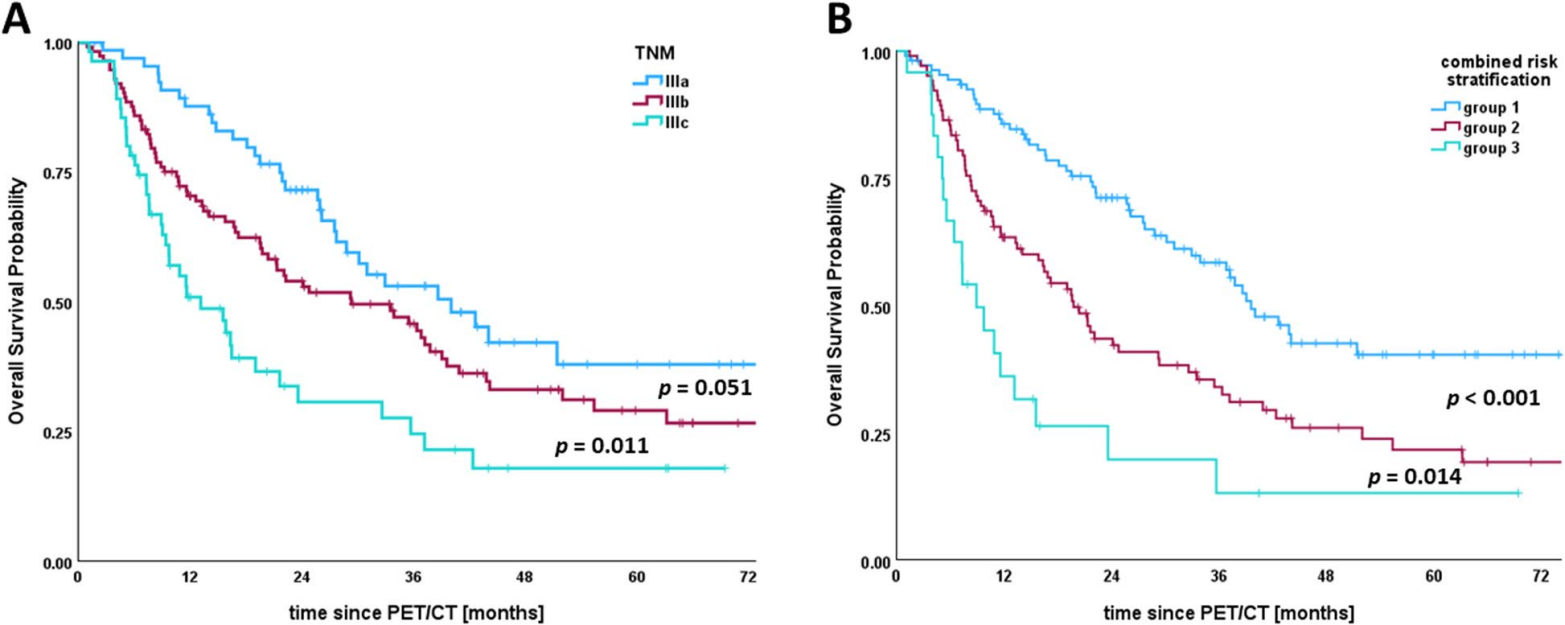
Kaplan-Meier plots for (**A**) TNM and (**B**) combined risk stratification (TNM + MTV) in patients with locally advanced non-small cell lung cancer receiving chemoradiotherapy. **A** Median overall survival for stages IIIa, IIIb, and IIIc were 40, 29, and 13 months, respectively (log rank *p* = 0.051 and *p* = 0.011). **B** Median overall survival for groups 1 (stage IIIa and stage IIIb with MTV < 48 ml), 2 (stage IIIb with MTV > 48 ml and stage IIIc with MTV < 105 ml), and 3 (stage IIIc with MTV > 105 ml) were 40, 20, and 9 months, respectively (log rank *p* = 0.001 and *p* = 0.014). Vertical lines indicate censoring

Including the stage-specific cut-off values ​​for MTV, we performed a subgroup analysis of each locally advanced stage IIIa-c. Therefore, Hazard Ratios (HR) for OS were calculated in six subgroups classified according to TNM and optimal cut-off values for MTV (Supplementary Table 3). The HRs of patients in stage IIIa with low MTV (< 45 ml), patients in stage IIIa with high MTV (> 45 ml), and patients in stage IIIb with low MTV (< 48 ml) demonstrated no significant difference from each other. Likewise, the HRs of patients in stage IIIb with high MTV (> 48 ml) and stage IIIc with low MTV (< 105 ml) demonstrated no significant difference from each other. Patients in stage IIIa, and patients in stage IIIb with low MTV (< 48 ml) were assigned to risk group (1) Patients in stage IIIb with high MTV (> 48 ml) and stage IIIc with low MTV (> 105 ml) were assigned to risk group (2) Patients in stage IIIc with high MTV (> 105 ml) were assigned to risk group (3) In the UICC staging system, the HRs of stage IIIa and stage IIIb showed no significant difference. In contrast, the HRs of risk groups 1, 2, and 3 showed significant differences for OS probability (Table 4). Compared to survival curves based on UICC stages (IIIa, IIIb, and IIIc), those based on the combined risk stratification (groups 1, 2, and 3) discriminated prognosis better (Fig. 5).

**Table 4:** Univariable Cox Proportional Hazards Regression Analyses for Overall Survival of traditional TNM and combined risk stratification

Variables	All patients			
	HR	95%	CI	*p* value
UICC				< 0.001
IIIa	reference category			
IIIb	1.519	0.999	2.310	0.050
IIIc	**2.612**	1.625	4.200	< 0.001
combined risk stratification (TNM + MTV)				< 0.001
1	reference category			
2	**2.012**	1.399	2.894	< 0.001
3	**3.898**	2.281	6.662	< 0.001

Bold numbers denote statistical significance

*Abbreviations:* *HR* Hazard Ratio, *CI* Confidence Interval

## Discussion

Clinical TNM staging currently is the main basis for decisions on therapeutic choices. However, in daily routine it is used with different intentions. To evaluate operability, a strong focus is commonly put on the N staging. Since it increases the likelihood of achieving R0 resection margins and microscopic tumor control, it is the main aim of curative surgical procedures. In contrast, curative local chemoradiotherapy is increasingly put into perspective with multimodal therapy approaches to address possible micrometastases by abscopal effects attributed to the immune system [4], raising the need for new staging parameters beyond TNM.

Our findings that the volumetric PET parameters MTV and TLG are significantly prognostic for OS in univariable Cox regression analyses are consistent with previous results reported by other study groups [9, 13, 15–21]. In the multivariable Cox regression analysis, both volumetric parameters were significant predictors of OS independent of substage, but did not remain significant in the multivariable Cox analyses of the separate cohorts, most likely due to low study group size or possible pre-selection bias for the two groups as discussed in the limitations later. Since TLG is defined as the product of MTV and SUVmean (normalized to body weight or lean body mass), both volumetric PET parameters highly correlate with each other and could not be included in the same multivariable regression model. For practical reasons, we have focused on MTV, as this parameter with its unit in milliliters is possibly easier to use in everyday clinical practice and is more relevant for radiation therapy.

When adjusted to TNM and MTV, the therapeutic strategy was not predictive of OS. We attribute this to our previous observation of significantly lower MTV in the surgical group and a pre-selection bias for surgical candidates: patients with higher MTV were more likely to be not suitable for surgery. In contrast to MTV, number of PET positive lesions, SUV_max_, and SUL_peak_ were not prognostic of OS, as supported by similar results in literature [9, 10, 16, 29]. In estimating prognosis of locally advanced NSCLC, MTV as a marker of tumor burden is superior to the pure number of tumor manifestations or traditional PET parameters, e.g. SUV_max_. Interestingly, Tan et al. reported a similar prognostic value of MTV combined with a parameter for tumor dissemination (Dmax) in patients with metastatic NSCLC [39]. In locally advanced disease stages, the benefit of this additional parameter yet remains unclear. However, we were able to show that not only tumor volume, but also the radius of the tumor volume MTV(r) were independent prognostic factors in disease stage III.

Locally advanced NSCLC treated with platinum-based concurrent chemoradiotherapy is associated with poor prognosis [1, 5]. In our study population, 28% of patients were alive after 5 years and median survival for inoperable patients was 28 months - the sad historical reality of the last decade. Modern treatment regimens in non-surgical candidates consist of a multimodal approach with chemoradiotherapy supported by targeted or immune modulatory therapy, improving patient prognosis [3, 5]. But also presenting medical imaging with new challenges, e.g. immunological abscopal effects, that demand new imaging parameters, e.g. MTV, for staging beyond traditional TNM [4, 7]. We found significantly higher metabolic tumor volumes in inoperable patients than in surgical candidates, and in more advanced stages, similar to observations in literature [14, 19, 21]. Its independent prognostic value in a multivariable model along with treatment decisions and other clinical variables, makes it an ideal parameter to collect in baseline studies and to monitor during therapy.

For the second aim of the study, we restricted our analyses to the subset of patients treated with chemoradiotherapy (*n* = 211) to reduce bias of heterogenous therapy approaches. Using ROC curve analysis we determined an optimal cut-off value of MTV = 45 ml for our cohort, similar to the 52 ml-cut-off identified by Lapa et al. who used an absolute threshold of SUV > 2.5 for MTV [17]. In contrast, other studies reported cut-off values between 20 and 30 ml in UICC stage III using absolute, relative and gradient-based threshold methods for determining MTV [15, 18, 19]. However, we could observe significantly different survival curves by our cut-off value for all patients treated by chemoradiotherapy. We were able to observe the same trend, when considering clinical and trial cohort separately, supporting the feasibility of liver background adjustment for MTV in trials and everyday clinical routine. For the purpose of additional prognostic information to TNM, MTV above physiological liver uptake could semi-automatically be obtained as part of the baseline assessment of PERCIST [32]. This could be a valuable additional stratification method in clinical trial analyses and might have impact of guiding extent and length of adjuvant therapies.

Regarding patients in stages IIIa-c, MTV was able to further sub stratify patients with stage-specific cut-off values, resulting in significant differences in median OS of approximately 12 months within substages. This aligns with the study of Lapa et al. who achieved similar results in dividing stages by MTV [17]. Finkle et al. stratified patients in stage IIIA by MTV into two categories and could prove that both subgroups did not differ from their adjacent categories IIB and IIIB regarding OS [19]. Since the subdivision of TNM stages IIIa-c by stage-specific MTV values ​​showed significant differences in OS, we decided to combine TNM and MTV. When stratified into three risk groups according to the combination TNM stage and MTV, the combination of TNM and MTV was able to better predict overall survival probability. Similar to previous observations in esophageal cancer [24], the assignment of risk groups according to a combination of TNM stage and MTV, more accurately discriminates disease prognosis in locally advanced NSCLC than TNM stage IIIa-c alone. Prospective trials on multimodal therapy concepts, including immune modulatory therapy, could benefit from the inclusion of MTV, particularly in a locally advanced stage with heterogeneous TNM [15, 18, 19].

One strength of the study certainly is the delineation method used for MTV above an individual liver background threshold, which was not assessed priorly for prognostication [33]. Another special aspect that PET data of more than 20 different institutions were used [37]. Current studies on this topic are mainly retrospective and involve single-center data [8, 16, 17, 25, 26, 29]. With only a few studies collecting data from two or more centers [10, 19, 20]. Numbers of patients mainly involve a small sample size, with commonly less than 300 patients [8, 10, 15–17, 19, 21, 27, 35]. With meticulous harmonization of the participating study centers, uniform use of the MTV is feasible even in large multi-center studies, as our study suggests.

The study has some limitations. First, the study population of the trial cohort underlies a pre-selection bias, since patients were deemed not suitable or not willing to undergo surgery. Although ECOG performance status of these patients was good, it remains unclear for the clinical cohort, since ECOG status was not documented. The target blood glucose level before administration of FDG was typically below 150 mg/dl. However, both in the trial cohort and the clinical cohort individual patients might have had higher blood glucose levels. However, also for initial validation of PERCIST criteria patients with blood glucose levels up to 200 mg/dl were allowed [32, 40]. In the trial group, patients received dose-escalated radiotherapy with doses between 60 and 74 Gy; however, documentation on patient individual doses in the clinical cohort was not available and therefore not used as a variable in the multivariable Cox regression analysis and its effect on the results remains unclear. Second, due to strict exclusion criteria number of patients is too low to make a generalized attempt on a new staging systems combining TNM, volumetric PET parameters and clinical variables. To address this concern, we suggest that future trials on PET/CT-based treatment planning or monitoring should include volumetric PET parameters as secondary outcomes. Third, we chose to obtain whole-body metabolic tumor volume and other PET parameters by a semi-automatic quantification method based on a threshold of liver background activity. Tumor uptake must be above the threshold (1.5*SULmean + 2*SD) to be measurable. Therefore, patients with tumor activity below liver threshold were excluded.

## Conclusions

Metabolic Tumor Volume determined by PET/CT is an excellent additional prognostic parameter in locally advanced NSCLC. It is an independent prognostic factor for OS similar to the UICC substages. However, its particular strength lies in the volumetric information as an addition to traditional TNM. A patient-specific liver background threshold for MTV proves to be advantageous in the context of multicenter studies. It could therefore make a significant contribution to assessing prognosis and guiding multimodal therapy strategies in future trials.

## Supplementary Information

Supplementary Material 1.

## Data Availability

No datasets were generated or analysed during the current study.

## Abbreviations

[18F]FDG: Fluorine-18-fluorodeoxyglucose
AC: Adenocarcinoma
CI: Confidence Interval
CRT: Chemoradiotherapy
HR: Hazard Ratio
IT: Immunotherapy
IQR: Interquartile Range
LCC: Large Cell Carcinoma
MTV: Metabolic Tumor Volume
MTV(r): radius of MTV
NSCLC: Non-small Cell Lung Cancer
OS: Overall Survival
PD-1: Programmed cell death 1
PD-L1: Programmed cell death-ligand 1
PET/CT: Positron Emission Tomography/ Computed Tomography
PERCIST: PET Response Criteria in Solid Tumor
PFS: Progression-free Survival
ROC: Receiver Operating Characteristic
SCC: Squamous Cell Carcinoma
SD: Standard Deviation
SULmean: mean Standardized Uptake Value corrected for Lean Body Mass
SULpeak: peak Standardized Uptake Value corrected for Lean Body Mass
SUVmax: maximum Standardized Uptake Value
TLG: Total Lesion Glycolysis
UICC: Union for International Cancer Control
VOI: Volume of Interest
