# Supplementary material for: Prognostic value of metabolic tumor volume on [18F]FDG PET/CT in addition to the TNM classification system of locally advanced non-small cell lung cancer
Source: Cancer Imaging. 2024 Dec 21;24:171. doi: 10.1186/s40644-024-00811-7 (PMC11662478; doi:10.1186/s40644-024-00811-7)
Supplement: Supplementary file 1 — Supplementary Material 1. [file 40644_2024_811_MOESM1_ESM.docx]

Supplementary Tables

| **Supplementary Table 1** | | | | | |  |
| --- | --- | --- | --- | --- | --- | --- |
| Multivariable Cox proportional Hazards Regression Analysis for Overall Survival | | | | | |  |
|  | Variables | All patients | | | |  |
|  |  | HR | 95% CI | | *p* value |  |
|  | Cohort^a^ | 1.078 | 0.623 | 1.864 | 0.789 |  |
|  | Age | **1.022** | 1.001 | 1.043 | 0.036 |  |
|  | Histology |  |  |  | 0.042 |  |
|  | AC | reference category | | | |  |
|  | SCC | **1.643** | 1.091 | 2.476 | 0.017 |  |
|  | NOS/ others | **1.694** | 1.002 | 2.864 | 0.049 |  |
|  | Treatment |  |  |  |  |  |
|  | Surgery | reference category | | | |  |
|  | Chemoradiotherapy | 1.396 | 0.62 | 3.139 | 0.420 |  |
|  | UICC stage |  |  |  | 0.002 |  |
|  | IIIa | reference category | | | |  |
|  | IIIb | 1.461 | 0.951 | 2.245 | 0.084 |  |
|  | IIIc | **2.437** | 1.479 | 4.017 | < 0.001 |  |
|  | MTV | **1.002** | 1.000 | 1.004 | 0.016 |  |
| Abbreviations: HR, Hazard Ratio; CI, Confidence Interval; NOS, not otherwise specified; MTV, metabolic tumor volume. a, trial cohort compared to clinical cohort. Bold values denote statistical significance. | | | | | |  |
|  |  |  |  |  |  |  |

| **Supplementary Table 2** | | | | | |  |
| --- | --- | --- | --- | --- | --- | --- |
| Multivariable Cox proportional Hazards Regression Analysis for Overall Survival | | | | | |  |
|  | Variables | All patients | | | |  |
|  |  | HR | 95% CI | | *p* value |  |
|  | Cohort^a^ | 1.046 | 0.601 | 1.821 | 0.873 |  |
|  | Age | **1.021** | 1.000 | 1.043 | 0.051 |  |
|  | Histology |  |  |  | 0.047 |  |
|  | AC | reference category | | | |  |
|  | SCC | **1.639** | 1.074 | 2.499 | 0.022 |  |
|  | NOS/ others | 1.718 | 0.999 | 2.955 | 0.050 |  |
|  | Treatment |  |  |  |  |  |
|  | Surgery | reference category | | | |  |
|  | Chemoradiotherapy | 1.445 | 0.642 | 3.254 | 0.374 |  |
|  | UICC stage |  |  |  | 0.002 |  |
|  | IIIa | reference category | | | |  |
|  | IIIb | 1.304 | 0.835 | 2.038 | 0.243 |  |
|  | IIIc | **2.386** | 1.437 | 3.962 | <0.001 |  |
|  | TLG | **1.000** | 1.000 | 1.000 | 0.027 |  |
| Abbreviations: HR, Hazard Ratio; CI, Confidence Interval; NOS, not otherweise specified; TLG, tumor lesion glycolysis. a, trial cohort compared to clinical cohort. Bold values denote statistical significance. | | | | | |  |
|  |  |  |  |  |  |  |

| **Supplementary Table 3** | | | | | |
| --- | --- | --- | --- | --- | --- |
| Univariable Cox Proportional Hazards Regression Analyses for Overall Survival | | | | | |
|  | Variables | All patients | | | |
|  |  | HR | 95% CI | | *p* value |
|  | TNM + MTV cut-off |  |  |  | *<0.001* |
|  | IIIa + MTV < 45 ml | reference category | | | |
|  | IIIa + MTV > 45 ml | 1.422 | 0.710 | 2.850 | 0.320 |
|  | IIIb + MTV < 48 ml | 1.109 | 0.559 | 2.201 | 0.768 |
|  | IIIb + MTV > 48 ml | 2.311 | 1.299 | 4.109 | 0.004 |
|  | IIIc + MTV < 105 ml | 2.387 | 1.210 | 4.709 | 0.012 |
|  | IIIc + MTV > 105 ml | 4.519 | 2.281 | 8.951 | < 0.001 |
| HR, Hazard Ratio; CI, Confidence Interval; MTV, metabolic tumor volume. | | | | | |
